# Traumatic brain injury derived pathological tau polymorphs induce the distinct propagation pattern and neuroinflammatory response

**DOI:** 10.1002/alz.089310

**Published:** 2025-01-03

**Authors:** Nicha Puangmalai, Nemil Bhatt, Alice Bittar, Cynthia Jerez, Nikita Shchankin, Rakez Kayed

**Affiliations:** ^1^ University of Texas Medical Branch, Galveston, TX USA

## Abstract

**Background:**

The misfolding and aggregation of the tau protein into neurofibrillary tangles constitute a central feature of tauopathies. Traumatic brain injury (TBI) has emerged as a potential risk factor, triggering the onset and progression of tauopathies. Our previous research revealed distinct polymorphisms in soluble tau oligomers originating from single versus repetitive mild TBIs. However, the mechanisms orchestrating the dissemination of TBI brain‐derived tau polymorphs (TBI‐BDTPs) remain elusive.

**Method:**

We explored whether TBI‐BDTPs could initiate pathological tau formation, leading to distinct pathogenic trajectories. Wild‐type mice were exposed to TBI‐BDTPs from sham, single‐blast (SB), or repeated‐blast (RB) conditions, and their memory function was assessed through behavioral assays at 2‐ and 8‐month post‐injection.

**Result:**

Our findings revealed that RB‐BDTPs induced cognitive and motor deficits, concurrently fostering the emergence of toxic tau aggregates within the injected hippocampus. Strikingly, this tau pathology propagated to cortical layers, intensifying over time. Importantly, RB‐BDTP‐exposed animals displayed heightened glial cell activation, NLRP3 inflammasome formation, and increased TBI biomarkers.

**Conclusion:**

Collectively, our results shed light on the intricate mechanisms underlying TBI‐BDTP‐induced tau pathology and its association with neuroinflammatory processes. This investigation enhances our understanding of tauopathies and their interplay with neurodegenerative and inflammatory pathways following traumatic brain injury.

